# Assessment of Diagnostic Competences With Standardized Patients Versus Virtual Patients: Experimental Study in the Context of History Taking

**DOI:** 10.2196/21196

**Published:** 2021-03-04

**Authors:** Maximilian C Fink, Victoria Reitmeier, Matthias Stadler, Matthias Siebeck, Frank Fischer, Martin R Fischer

**Affiliations:** 1 Institute for Medical Education University Hospital, LMU Munich Munich Germany; 2 Department of Psychology Ludwig-Maximilians-Universität München Munich Germany; 3 Munich Center of the Learning Sciences Ludwig-Maximilians-Universität München Munich Germany

**Keywords:** clinical reasoning, medical education, performance-based assessment, simulation, standardized patient, virtual patient

## Abstract

**Background:**

Standardized patients (SPs) have been one of the popular assessment methods in clinical teaching for decades, although they are resource intensive. Nowadays, simulated virtual patients (VPs) are increasingly used because they are permanently available and fully scalable to a large audience. However, empirical studies comparing the differential effects of these assessment methods are lacking. Similarly, the relationships between key variables associated with diagnostic competences (ie, diagnostic accuracy and evidence generation) in these assessment methods still require further research.

**Objective:**

The aim of this study is to compare perceived authenticity, cognitive load, and diagnostic competences in performance-based assessment using SPs and VPs. This study also aims to examine the relationships of perceived authenticity, cognitive load, and quality of evidence generation with diagnostic accuracy.

**Methods:**

We conducted an experimental study with 86 medical students (mean 26.03 years, SD 4.71) focusing on history taking in dyspnea cases. Participants solved three cases with SPs and three cases with VPs in this repeated measures study. After each case, students provided a diagnosis and rated perceived authenticity and cognitive load. The provided diagnosis was scored in terms of diagnostic accuracy; the questions asked by the medical students were rated with respect to their quality of evidence generation. In addition to regular null hypothesis testing, this study used equivalence testing to investigate the absence of meaningful effects.

**Results:**

Perceived authenticity (1-tailed *t*_81_=11.12; *P*<.001) was higher for SPs than for VPs. The correlation between diagnostic accuracy and perceived authenticity was very small (*r*=0.05) and neither equivalent (*P*=.09) nor statistically significant (*P*=.32). Cognitive load was equivalent in both assessment methods (*t*_82_=2.81; *P*=.003). Intrinsic cognitive load (1-tailed *r*=−0.30; *P*=.003) and extraneous load (1-tailed *r*=−0.29; *P*=.003) correlated negatively with the combined score for diagnostic accuracy. The quality of evidence generation was positively related to diagnostic accuracy for VPs (1-tailed *r*=0.38; *P*<.001); this finding did not hold for SPs (1-tailed *r*=0.05; *P*=.32). Comparing both assessment methods with each other, diagnostic accuracy was higher for SPs than for VPs (2-tailed *t*_85_=2.49; *P*=.01).

**Conclusions:**

The results on perceived authenticity demonstrate that learners experience SPs as more authentic than VPs. As higher amounts of intrinsic and extraneous cognitive loads are detrimental to performance, both types of cognitive load must be monitored and manipulated systematically in the assessment. Diagnostic accuracy was higher for SPs than for VPs, which could potentially negatively affect students’ grades with VPs. We identify and discuss possible reasons for this performance difference between both assessment methods.

## Introduction

### Performance-Based Assessment With Standardized Patients and Virtual Patients

Since the turn of the millennium, performance-based assessment has become a mandatory part of medical licensure examinations in various countries [1], complementing traditional assessment formats, such as text vignettes, with methods including standardized patients (SPs) and simulated virtual patients (VPs). SPs have been used for performance-based assessment in health care since the 1960s [2]. However, VPs have only recently become more widely employed in this domain [3].

The term SPs refers to (trained) actors or real former patients who act as if they display symptoms of a disease [4]. Usually, students encounter several SPs in assessment settings to reliably measure clinical variety [5]. Performance is then scored by a trained faculty member or the SPs themselves using a rating scheme. Although we will elaborate on the specific features used for this assessment method later, it should be noted here that organizing an assessment with SPs is relatively resource intensive [6].

VPs are a type of computer simulation and typically include an authentic model of a real-world situation that can be manipulated by the participant [7]. VPs can use avatars or realistic videos with SPs as stimuli and offer varying degrees of interaction [8]. Moreover, assessment through VPs can take place automatically, and a recent study showed that such an automatic assessment corresponds well to ratings from clinician-educators [9]. The production of authentic VPs can frequently produce considerable costs above $10,000 [10]. Although the initial production of VPs is often more resource intensive than organizing SPs, this assessment method is then permanently available and fully scalable to a large audience.

Next, we summarize a conceptual framework. This framework provides, on the one hand, a precise operationalization of diagnostic competences. On the other hand, the framework includes a research agenda that summarizes essential moderators of performance that should be examined systematically in research on simulation-based assessment.

### A Framework for the Assessment of Diagnostic Competences With Simulations

The framework developed by Heitzmann et al [10] to facilitate diagnostic competences with simulations operationalizes diagnostic competences in assessment settings as a disposition. This disposition encompasses the components of diagnostic knowledge, diagnostic quality, and diagnostic activities. Diagnostic knowledge includes conceptual and strategic knowledge [11]. Conceptual knowledge encompasses concepts and their relationships. Strategic knowledge comprises possible avenues and heuristics in diagnosing. Diagnostic quality consists of components’ diagnostic accuracy and efficiency that can serve as major outcome measures in empirical studies. Diagnostic activities entail the actions of persons assessed during the diagnostic process, such as evidence generation by asking questions in history taking. The framework proposes that context is an important moderator in assessment. Therefore, more research on the effects of the assessment methods SPs and VPs seems to be warranted. A meta-analysis on simulation-based learning of complex skills [12] added to this framework that authenticity should also be explored as an important moderator in assessment and learning. Similarly, a meta-analysis on instructional design features in simulation-based learning indicated that certain types of cognitive load could be detrimental to performance [13]. Therefore, it could be fruitful to explore the relationship between cognitive load and diagnostic competences within SP and VP assessments.

### Perceived Authenticity and Diagnostic Competences With SPs and VPs

There is a multitude of conceptualizations of authenticity. In our study, we focus on *perceived authenticity* [14] because this concept can be assessed entirely internally by learners’ judgment. Other related concepts such as *thick authenticity* [15] and *fidelity* [16] can, at least to some extent, also be determined externally.

According to a factor analysis by Schubert et al [14], perceived authenticity—sometimes also called presence—comprises the facets of realness, involvement, and spatial presence. Realness describes the degree to which a person believes that a situation and its characteristics resemble a real-life context [14]. Involvement is defined as a feeling of cognitive immersion and judgment that a situation has personal relevancy [17]. Spatial presence denotes the feeling of physical immersion in a situation [14]. SPs are considered highly authentic because they are carefully trained to realistically portray symptoms and allow for natural interactions [18]. Empirical studies support this claim, reporting high values of perceived authenticity for SPs [19,20]. VPs also received rather high perceived authenticity scores in empirical studies [21] but lacked some of the features that may make SPs particularly authentic, such as high interactivity in oral conversations. Thus, VPs could potentially evoke lower perceived authenticity than SPs. Findings on the effect of authenticity on diagnostic competences are mixed. On the one hand, it has been argued that higher authenticity is associated with higher engagement and better performance [22]. On the other hand, literature reviews [23,24] that compared the relationship between perceived authenticity and clinical performance in simulation-based learning only reported minimal effects of authenticity. In addition, an empirical study [25] showed that above a certain threshold, further increases in perceived authenticity do not improve diagnostic accuracy.

### Cognitive Load and Diagnostic Competences With SPs and VPs

Cognitive load theory posits that performance can be inhibited through high situational demands that stress working memory and attention [26]. The cognitive load consists of the following 3 different facets [27]: *Intrinsic* load results from the interplay between certain topics and materials and the assessed person’s expertise. *Extraneous load* is created exclusively by characteristics of the assessment environment that strain memory and attention without being necessary for performance. *Germane load* refers to the cognitive load created through the assessed person’s cognitive processes, including schema construction and abstraction. Intrinsic and extraneous cognitive loads are considered additive and can inhibit performance in complex tasks [27]. Germane load, however, is theorized to bolster performance [27]. A few primary studies from medical education have already contrasted the cognitive load of different assessment methods and reported their relationship with diagnostic competences. Dankbaar et al [28] demonstrated that intrinsic and germane cognitive loads were higher for a group learning emergency skills with a simulation game than for a group learning with a text-based simulation. Extraneous load did not differ between these groups, and none of the groups differed in performance. Haji et al [29] compared surgical skills training with less complex and more complex simulation tasks. The total cognitive load was higher in the more complex simulation than in the less complex simulation, and cognitive load was negatively associated with performance. As a result of these findings, we can conclude that SPs and VPs generally do not differ in different facets of cognitive load if the assessment methods are of equal complexity, and the main characteristics related to the facets are similar. The literature summarized earlier also shows that intrinsic and extraneous cognitive loads are negatively associated with diagnostic competences.

### Assessment Method and Diagnostic Competences

Before we discuss diagnostic accuracy and evidence generation—2 important aspects of diagnostic competences—it should be noted that diagnostic competences are only a part of the broader concept of clinical reasoning. Clinical reasoning emphasizes the process of diagnosing and encompasses the full process of making clinical decisions, including the selection, planning, and reevaluation of a selected intervention [30]. In line with the conceptual framework by Heitzmann et al [10] for facilitating diagnostic competences, *diagnostic accuracy* denotes the correspondence between the learner’s diagnoses and the solutions determined by experts for the same cases. According to this framework, *evidence generation* (ie, actions related to the gathering of data in a goal-oriented way) is also an important quality criterion for the diagnostic process and a crucial aspect of diagnostic competences.

#### Diagnostic Accuracy

Currently, there are only a few studies in the health care domain that contrast assessments using VPs and SPs directly in one experiment. Edelstein et al [1] investigated assessments with SPs and computer-based case simulations in advanced medical students using a repeated measures design. A moderate positive correlation was found between diagnostic accuracy in the two assessment formats that used different cases. Guagnano et al [31] examined SPs and computer-based case simulations in a medical licensing exam. Participants first completed the computer-based case simulations and then completed the SPs. The two assessment methods correlated positively with each other. Hawkins et al [32] compared the assessment of patient management skills and clinical skills with SPs and computer-based case simulations in a randomized controlled trial. Participating physicians completed both assessment methods, and a positive correlation of diagnostic accuracy with both assessment methods was reported. Outside the health care domain, a meta-analysis of studies from different domains reported a robust modality effect for students in problem-solving tasks. Students who solved problems presented in the form of illustrations accompanied by text were more successful than students who solved problems presented merely in text form [33]. Similarly, it seems reasonable to assume that one assessment method could lead to higher diagnostic accuracy than the other assessment method because of its different characteristics. The described findings from the health care domain tentatively indicate that SPs and VPs could result in relatively equivalent diagnostic accuracy. Such a finding would contradict the modality effect reported in other domains.

#### Evidence Generation

Comparable empirical studies on evidence generation for SPs and VPs are lacking. Nevertheless, we can assume that the quantity of evidence generation should be higher for SPs than for VPs. The main reason for this is that students can ask questions of SPs more quickly orally than by selecting questions from a menu of options with VPs. Apart from this difference in evidence generation between the 2 assessment methods, the relationships between evidence generation and diagnostic accuracy are interesting. The relationship between the quantity of evidence generation and diagnostic accuracy is relatively complex. The ideal amount of evidence generation may depend strongly on the case difficulty, the diagnostic cues contained in the evidence, and learner characteristics. For these reasons, the framework by Heitzmann et al [10] for facilitating diagnostic competences argues that the sheer quantity of evidence generation is not a dependable quality criterion for the diagnostic process. However, the quality of evidence generation is hypothesized by Heitzmann et al [10] to be a rather dependable quality criterion for the diagnostic process. This agrees with the literature, as we know from studies on SPs using observational checklists that the quality of evidence generation is positively associated with diagnostic accuracy [34]. Moreover, one study with specialists in internal medicine and real patients demonstrated that asking specific questions in history taking correlated positively with clinical problem solving [35].

### Study Aim, Research Questions, and Hypotheses

We aim to compare the perceived authenticity, cognitive load, and diagnostic competences in SPs and VPs. We also aim to examine the relationships of perceived authenticity, cognitive load, and quality of evidence generation with diagnostic accuracy. Thus, we address the following 3 research questions: To what extent does perceived authenticity differ across the 2 assessment methods, and how is it associated with diagnostic accuracy (RQ1)? We hypothesize that SPs induce higher perceived authenticity than VPs (H1.1). Moreover, we expect to be able to demonstrate with equivalence tests for correlations (given in the *Statistical Analyses* section) that perceived authenticity is not associated meaningfully with diagnostic accuracy (H1.2). Next, is cognitive load equivalent for SPs and VPs, and how is it related to diagnostic accuracy (RQ2)? We assume to find equivalent cognitive load for SPs and VPs (H2.1). Moreover, we expect that intrinsic and extraneous loads are negatively related to diagnostic accuracy (H2.2-H2.3). To what extent are the diagnostic competences components diagnostic accuracy, quantity of evidence generation, and quality of evidence generation equivalent or differ for SPs and VPs, and how are they related to each other (RQ3)? We hypothesize that SPs and VPs evoke equivalent diagnostic accuracy (H3.1). In addition, we assume that the quantity of evidence generation is higher for SPs than for VPs (H3.2). We also expect that the quality of evidence generation is positively related to diagnostic accuracy (H3.3).

## Methods

### Participant Characteristics and Sampling Procedures

A sample of 86 German medical students (with a mean age of 26.03 years, SD 4.71) made up the final data set. This sample consisted of 63% (54/86) females and 37% (32/86) males. Medical students in years 3-6 of a 6-year program with a good command of German were eligible. Medical students in years 3-5 (44/86, 51%) were considered novices, as they were still completing the clinical part of the medical school. Medical students in year 6 (42/86, 49%) were regarded as intermediates as they had passed their second national examination and worked full time as interns in a medical clinic or practice. We provide a detailed overview of participant characteristics across all conditions and a CONSORT (Consolidated Standards of Reporting Trials)–style diagram of participant flow in Multimedia Appendix 1.

We collected data from October 20, 2018, to February 20, 2019, in the medical simulation center of the University Hospital, LMU Munich. We recruited participants via on-campus and web-based advertising. Participants were randomly assigned to conditions by the first author by drawing a pin code to log in to an electronic learning environment without knowing the condition assigned to the pin. In the final data collection sessions, the conditions were filled by the first author with random participants from specific expertise groups (novices vs intermediates). This procedure was applied to achieve a comparable level of expertise in all conditions. As expected, the proportion of participants from different expertise groups did not differ across conditions (*χ*²_3_=0.2; *P*=.99).

### Research Design

The study used a repeated measures design with assessment method (SPs vs VPs) as the key factor. In addition, we varied the between-subjects factor case group (CG) order and assessment method order. In total, students encountered 6 different cases. We provide an overview of the experiment in Table 1. Details of the succession through cases and medical content in the experimental conditions are provided in Table 2. We attempted to ensure similar topics and difficulty for both CGs by conducting an expert workshop and adapting cases based on the experts’ feedback as part of creating the experimental materials.

**Table 1:** General overview of the experiment.

Part of the experiment	Activity or test	Duration (min)
Pretest	Briefing	10
	Conceptual knowledge test	40
	Strategic knowledge test	40
Break	—^a^	10
Assessment phase I (cases 1-3)	VPs^b^ or SPs^c^	70
Break and change of modality	—	5
Assessment phase II (cases 4-6)	VPs or SPs	70
Posttest and debriefing	Working memory test	15
	End-debriefing	5

^a^No activity or test takes place.

^b^VP: virtual patient.

^c^SP: standardized patient.

**Table 2:** Succession through cases and medical content in the experimental conditions^a,b^.

Cases	Condition 1A	Condition 1B	Condition 2A	Condition 2B
1-3	CG^c^ A (SPs^d^)	CG B (VPs^e^)	CG B (SPs)	CG A (VPs)
4-6	CG B (VPs)	CG A (SPs)	CG A (VPs)	CG B (SPs)

^a^Case group A: (1) pulmonary embolism with lymphoma, (2) congestive heart failure with atrial fibrillation, and (3) hyperventilation tetany caused by a panic attack.

^b^Case group B: (1) pulmonary embolism with coagulation disorder, (2) community-acquired pneumonia, and (3) hypertrophic obstructive cardiomyopathy.

^c^CG: case group.

^d^SP: standardized patient.

^e^VP: virtual patient.

### Procedure and Materials

Participants completed a pretest of conceptual knowledge and strategic knowledge at the beginning of the experiment. Afterward, participants took part in the assessment phase, solving the first 3 cases with SPs and the next 3 cases with VPs or vice versa. All cases were drafted by a specialist in general practice and evaluated positively by an expert panel. The cases were not adapted from real clinical cases but based on cases from textbooks and symptoms reported in guidelines. A short familiarization phase preceded each assessment phase and included a motivational scale. For all cases in both assessment methods, assessment time was held constant at 8 minutes and 30 seconds for history taking and 5 minutes for writing up a diagnosis for the case in an electronic patient file. At the end of the experiment, participants were debriefed. A more detailed overview of the procedure can be found in Multimedia Appendix 2.

**Figure 1:**
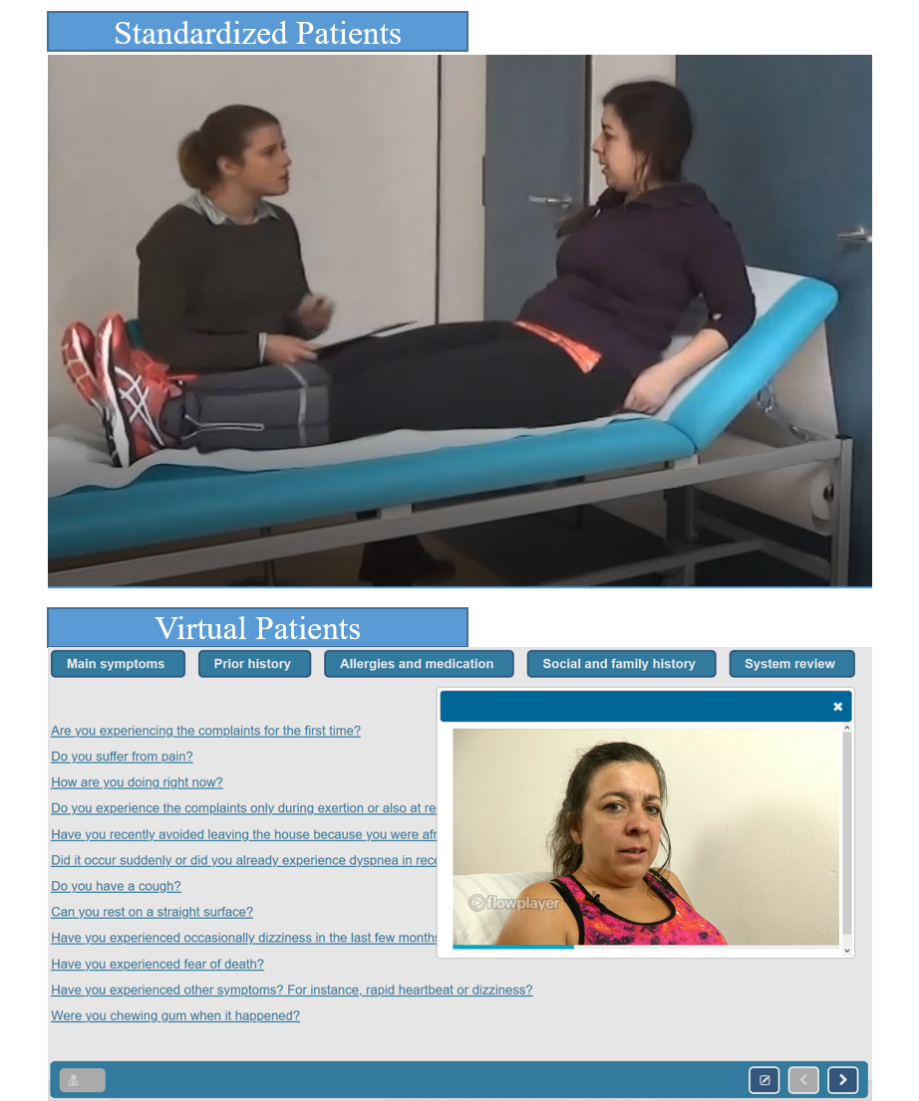
History-taking with standardized patients and virtual patients.

Assessment with SPs was conducted in a simulated emergency room. All SPs were (semi-) professional actors who were financially compensated; most had previous experience working in an SP program. All SPs were extensively trained by an acting coach and a physician, memorized their symptoms and scripts, and were not aware of their patient’s diagnosis. Participants first received prior information (eg, electrocardiogram and lab results) and presentation of the chief complaint for each case. Next, participants formulated and asked questions independently, and the SPs responded. The interaction was recorded on a video. After each case, the participants completed a patient file, including measures of diagnostic accuracy and other scales. A screenshot of this assessment method is provided in Figure 1.

The assessment with the VPs was carried out in a simulated assessment environment in a computer room. First, participants received prior information and a video with a chief complaint for each case. The participants then selected questions independently from a menu with up to 69 history-taking questions. The VP’s answer was streamed as a video, including a recorded response by an actor. After each case, the participants completed a patient file, including a measure of diagnostic accuracy and other scales. A screenshot of this assessment method is provided in Figure 1.

The VPs, patient file, and other measures were implemented in the electronic assessment environment CASUS [36]. The questions provided for the VPs were based on a structural and topical analysis of history-taking forms by Bornemann [37] and are displayed in Multimedia Appendix 3. According to this analysis, physician questions in history taking can fall under the 5 categories of main symptoms, prior history, allergies and medication, social and family history, and system review. Participants with SPs received empty history-taking forms for all cases and time to formulate possible history-taking questions during the familiarization phase, at which point participants in the VPs only read all questions from the menu. Without this additional structuring support in the SP condition, the participants in the VP condition would have received additional support in the form of a list of questions in the menu.

### Measures and Covariates

#### Perceived Authenticity

Perceived authenticity was operationalized as a construct with the 3 dimensions of realness, involvement, and spatial presence [14]. All 3 authenticity scales used a 5-point scale ranging from (1) *disagree* to (5) *agree* and were taken from multiple validated questionnaires [14,38-40]. The items were slightly adapted to simulation-based assessment and are included in Multimedia Appendix 4. A combined score for all 3 dimensions was built by calculating the mean. This scale achieved a reliability of Cronbach α=.88.

#### Cognitive Load

The cognitive load scale by Opfermann [41] used in this study assessed the extraneous cognitive load with 3 items and germane and intrinsic cognitive loads with 1 item each. A 5-point scale from (1) *very easy*, (2) *rather easy*, (3) *neutral*, (4) *rather hard*, to (5) *very hard* was used. The scale is included in Multimedia Appendix 4. A combined score for all 3 facets was built by calculating the mean. This scale achieved a reliability of Cronbach α=.88.

#### Motivation, Diagnostic Knowledge, and Other Control Variables

We assessed motivation as a control variable because it could differ between assessment methods and potentially affect performance. The expectancy component of motivation was assessed with a 4-item, 7-point scale adapted from Rheinberg et al [42]. The motivation expectancy scale ranged from (1) strongly disagree to (7) strongly agree*.* The value component of motivation was measured with a 4-item, 5-point scale based on a questionnaire by Wigfield [43]. The motivation value scale ranged from (1) strongly disagree to (5) strongly agree. The full scales are provided in Multimedia Appendix 4. Diagnostic knowledge was also measured in this study but later not taken into account in the analyses because it was similar in VPs and SPs because of the repeated measures design. We measured diagnostic knowledge using a conceptual and strategic knowledge test. Both types of knowledge have been identified as predictors of clinical reasoning [44]. The maximum testing time was set to 40 minutes per test. More details on both diagnostic knowledge tests are reported in Multimedia Appendix 4. Apart from this, demographic data were collected, including participants’ sex, age, and expertise (year of medical school).

### Diagnostic Competences

#### Diagnostic Accuracy

Diagnostic accuracy was assessed based on the answer to the prompt “Please choose your final diagnosis after history taking” from a long menu containing 239 alternative diagnoses. Two physicians created a coding scheme for scoring diagnostic accuracy in all cases (Multimedia Appendix 4). To do that, the physicians rated all 239 alternative diagnoses for all cases and resolved the disagreements until they reached full agreement. One of the physicians was a specialist in general practice who also drafted the cases. The other physician was a board-certified doctor familiar with medical assessment through her dissertation. The latter physician, who is also the second author of this paper, then scored diagnostic accuracy based on the coding scheme: 1 point was allocated for the designated correct answer, 0.5 point for a partially correct answer, and 0 point for an incorrect answer. Due to having only 1 rater to score the diagnostic accuracy with the comprehensive coding scheme, a reliability estimate cannot be reported. However, this is also not necessary because the exact diagnostic accuracy score for all selectable diagnoses included in the electronic assessment environment was determined upfront in the coding scheme.

#### Evidence Generation

The second author classified the quality of evidence generation by determining the essential questions relevant for the correct diagnosis for each VP case (the coding scheme is given in Multimedia Appendix 4). This process took part before looking at the experimental data. All solutions were discussed with a specialist in general practice, and all disagreements were resolved. Student assistants transcribed all utterances recorded in the videos of the SP encounters, and the electronic assessment environment stored all selected questions during the VP encounters. The *R* scripts automatically classified the log data from the VPs using the coding scheme. Student assistants had no medical background and were trained by the second author to code the transcripts from the SP encounters. This task mainly implied recognizing the intent of history-taking questions and linking them, if possible, to the most similar question in the coding scheme. After training the raters, 20% of this complex and extensive SP data were coded by 2 raters to check interrater agreement. This data set encompassed SP data from 18 of the 86 participants of our study with all three SP cases in which the participants took part. Fleiss κ=0.74 demonstrated that agreement was substantial, and the rest of the data were coded by the same raters individually. The score for quantity of evidence generation corresponded to the total number of questions posed for each case. To calculate the score for quality of evidence generation for each case, we counted the number of relevant questions posed and divided this score by the number of relevant questions that could potentially be posed.

#### Scale Construction

Diagnostic accuracy and evidence generation scales for each assessment method and combining the 2 methods were built by calculating the mean of the included cases. Case 1 in CS A was excluded from all analyses because of high difficulty (mean diagnostic accuracy 0.05, SD 0.18).

### Statistical Analyses

This study answers the proposed research questions using traditional null hypothesis significance testing (NHST) and equivalence testing. In contrast to NHST, equivalence testing can be used to investigate “whether an observed effect is surprisingly small, assuming that a meaningful effect exists in the population” [45]. For this type of test, first, the smallest effect size of interest, that is, the threshold for a meaningful effect, is specified based on the literature. The null hypothesis that the effect is more extreme than the smallest effect size of interest is then investigated. To do this, 2 separate 1-sided tests (TOST; eg, *t* tests) are conducted [46]. These tests examine whether the observed effect is more extreme than the specified smallest effect size of interest. If both 1-sided tests are significant, the null hypothesis that there is a meaningful effect that is more extreme than the smallest effect size of interest is rejected. Thus, equivalence is supported. For more convenient reporting, only the *t* test with a higher P value is reported. In cases in which equivalence cannot be supported, NHST is performed for follow-up analyses.

All statistical analyses were performed using *R* version 3.6.1 [47]. The TOST procedure and the corresponding package TOSTER [45] were used to conduct the equivalence tests. In all statistical analyses, the alpha level was set to 5%; 1-tailed tests were used where applicable. The Bonferroni-Holm method [48] was used to correct P values for multiple comparisons in post hoc and explorative tests.

For all equivalence tests, the smallest effect size of interest was determined based on the discussed literature. For H1.2 and related post hoc tests, the smallest effect size of interest was set to be more extreme than *r*=±0.20, which corresponds to the effect size of small but meaningful correlations typically encountered in the social sciences [49]. For H2.1 and related post hoc tests, a meaningful effect was determined as an effect of Cohen *d*=0.35. This effect size lies between a small effect (Cohen *d*=0.20) and a medium effect (Cohen *d*=0.50) [49] and occurs frequently in the social sciences. For H3.1, we determined that a meaningful effect exists in the case of a difference of ±0.125 points in diagnostic accuracy. This was based on supposing a pass cutoff of 0.50 for diagnostic accuracy (ranging from 0 to 1) and setting 4 equal intervals for the hypothetical passing grades A-D.

### Power Analysis

We conducted a priori power analysis for dependent samples *t* tests (H1.1 and H3.2). This power analysis was based on a small to medium effect of Cohen *d*=0.30, 2-tailed testing, an error probability of 5%, and 80% power, resulting in a targeted sample of 90 participants. Moreover, we carried out a priori power analyses for 1-tailed correlations with *r*=±0.25, an error probability of 5%, and 80% power (H2.2-H2.3 and H3.3). This power analysis resulted in a planned sample size of 95 participants. A post hoc power analysis for the main equivalence test (H3.1) with 86 participants, the observed effect of Cohen *d*=0.26, and an error probability of 5% resulted in a power of 78%. All power analyses were conducted using G*Power software [50].

## Results

### Descriptive Statistics and Analysis of Control Variables

Descriptive statistics are provided in Table 3. The perceived authenticity variables were rated as very high for SPs and relatively high for VPs. Cognitive load variables were reported to be moderate in both assessment methods. The average diagnostic accuracy was medium. The quantity of evidence generation was higher for SPs than for VPs. The quality of evidence generation was medium for both assessment methods. Motivational variables were rated rather highly for both SPs and VPs. A post hoc comparison showed that the value aspect of motivation was higher for SPs than for VPs (2-tailed t_83_=2.89; *P*=.01; Cohen *d*=0.31), whereas the expectancy aspect did not differ between assessment methods (2-tailed t_83_=0.44; *P*=.66; Cohen *d*=0.05). Participants demonstrated slightly above medium performance on the conceptual and strategic knowledge tests. Multimedia Appendix 5 provides an additional visualization of the results using boxplots and bee swarm plots.

**Table 3:** Descriptive statistics.

Variable		Both methods, mean (SD)	SPs^a^, mean (SD)	VPs^b^, mean (SD)
**Perceived authenticity^c^**		3.62 (0.67)	4.02 (0.67)	3.23 (0.84)
	Realness^c^	3.71 (0.79)	4.13 (0.74)	3.28 (1.07)
	Involvement^c^	3.82 (0.66)	4.03 (0.73)	3.61 (0.83)
	Spatial presence^c^	3.35 (0.80)	3.89 (0.83)	2.80 (1.05)
**Cognitive load^c^**		2.88 (0.61)	2.88 (0.74)	2.90 (0.69)
	Intrinsic load^c^	3.18 (0.68)	3.20 (0.78)	3.14 (0.80)
	Extraneous load^c^	2.84 (0.65)	2.82 (0.79)	2.87 (0.76)
	Germane load^c^	2.74 (0.76)	2.73 (0.88)	2.76 (0.84)
**Diagnostic competences**				
	Diagnostic accuracy^d^	0.46 (0.18)	0.51 (0.28)	0.41 (0.24)
	Quantity of evidence generation	22.26 (4.88)	29.01 (8.03)	17.34 (4.21)
	Quality of evidence generation^d^	0.40 (0.11)	0.37 (0.18)	0.43 (0.13)
**Control variables**				
	Motivation expectancy aspect^e^	5.07 (0.91)	5.10 (0.88)	5.05 (1.08)
	Motivation value aspect^c^	4.44 (0.51)	4.54 (0.54)	4.34 (0.67)
	Conceptual knowledge^d^	0.65 (0.14)	—^f^	—
	Strategic knowledge^d^	0.66 (0.15)	—	—

^a^SP: standardized patient.

^b^VP: virtual patient.

^c^Scale range: 1-5.

^d^Scale range: 0-1.

^e^Scale range: 1-7.

^f^Knowledge was assessed before taking part in SPs and VPs.

### Perceived Authenticity and Diagnostic Accuracy (RQ1)

A paired sample *t* test demonstrated that in line with hypothesis H1.1, perceived authenticity was considered higher for SPs than VPs in terms of the combined score (1-tailed t_81_=11.12; *P*<.001; Cohen *d*=1.23). Post hoc tests showed that this was also the case for realness (t_80_=8.83; *P*<.001; Cohen *d*=0.98), involvement (t_81_=4.60; *P*<.001; Cohen *d*=0.51), and spatial presence (t_79_=10.65; *P*<.001; Cohen *d*=1.19). Our expectation in H1.2 was that perceived authenticity would not be meaningfully associated with diagnostic accuracy. The TOST procedure for correlations showed that the relationship between diagnostic accuracy and the combined perceived authenticity score (*r=*0.05; *P*=.09) was outside the equivalence bounds of a meaningful effect of *r*=±0.20. Post hoc equivalence tests demonstrated that this also holds for the relationship of diagnostic accuracy with realness (*r=*0.03; *P*=.06), involvement (*r=*0.07; *P*=.11), and spatial presence (*r=*0.05; *P*=.08). Reanalyzing these correlations with regular 1-tailed NHST tests also yielded nonsignificant results for the combined score (*P*=.32), realness (*P*=.39), involvement (*P*=.28), and spatial presence (*P*=.33). These results mean that there is neither evidence for the absence of meaningful correlations nor evidence for significant correlations. These inconclusive findings may stem from the lack of statistical power because of the relatively small sample size [45].

### Cognitive Load and Diagnostic Accuracy (RQ2)

We hypothesized in H2.1 that we would find equivalent cognitive load scores for SPs and VPs. Equivalence testing with the TOST procedure for paired samples indicated that for both assessment methods, the scores for combined cognitive load (t_82_=2.81; *P*=.003) were significantly within the equivalence bounds of an effect of Cohen *d*=0.35. Adjusted post hoc equivalence tests showed that this is also the case for intrinsic load (t_82_=−2.47; *P*=.008), extraneous load (t_82_=2.55; *P*=.01), and germane load (t_82_=2.64; *P*=.01). We expected in H2.2-H2.3 to uncover negative correlations between diagnostic accuracy and intrinsic cognitive load and extraneous load. As assumed, intrinsic cognitive load (1-tailed *r=−*0.30; *P*=.003) and extraneous load (1-tailed *r=−*0.29; *P*=.003) correlated negatively with the combined score for diagnostic accuracy. Adjusted explorative follow-up analyses showed that germane load (*r=−*0.25; *P*=.010) and the total score for cognitive load (*r=−*0.31; *P*=.004) also correlated negatively with the combined score for diagnostic accuracy.

### Assessment Method and Diagnostic Competences (RQ3)

#### Diagnostic Accuracy

In H3.1, we hypothesized finding equivalent diagnostic accuracy scores for SPs and VPs. H3.1 was first examined by applying a paired samples TOST procedure. According to our data, we cannot reject hypothesis H3.1 that a difference in diagnostic accuracy of at least ±0.125 points (1 grade) exists between the 2 assessment methods (t_85_=−0.60; *P*=.28). A follow-up 3-way mixed design analysis of variance demonstrated that neither the CG order nor the assessment method order (*F*_3,82_=2.49; *P*=.12; η^2^=0.03, respectively, *F*_3,82_=0.02; *P*=.88; η^2^=0.01) had a significant effect on diagnostic accuracy. The assessment method itself, however, had a significant main effect (*F*_3,82_=6.30; *P*=.01; η^2^=0.07), indicating that diagnostic accuracy was higher for SPs than for VPs. The finding that diagnostic accuracy was higher for SPs than for VPs also corresponds to the result of a paired sample *t* test (2-tailed t_85_=2.49; *P*=.01; Cohen *d*=0.27).

#### Evidence Generation

H3.2 that students display an increased quantity of evidence generation with SPs than with VPs was supported (1-tailed t_69_=12.26; *P*<.001; Cohen *d*=1.47). However, in an explorative follow-up analysis, we found no evidence that the *quantity* of evidence generation was related to diagnostic accuracy (1-tailed *r=*0.11; *P*=.15). This finding holds equally for SPs (*r=−*0.09; *P*=.76) and VPs (*r=−*0.10; *P*=.82). Moreover, H3.3 that the *quality* of evidence generation is positively related to diagnostic accuracy in both assessment methods was not supported (1-tailed *r=*0.18; *P*=.05). Corrected post hoc analyses showed, however, that the quality of evidence generation was positively related to diagnostic accuracy for VPs (*r=*0.38; *P*<.001); this finding did not hold for SPs (*r=*0.05; *P*=.32). Additional post hoc exploratory analyses revealed that the quality of evidence generation was higher for VPs than for SPs (2-tailed t_74_=–2.47; *P*=.02; Cohen *d*=0.29).

## Discussion

### Principal Findings

With regard to perceived authenticity, our results showed that SPs and VPs achieved high scores on all 3 dimensions of realness, involvement, and spatial presence. Despite this high level of perceived authenticity in both assessment methods, perceived authenticity was higher for SPs than for VPs on all 3 dimensions. This finding is in line with the literature, which has long claimed that SPs achieve a very high level of perceived authenticity [18-20]. Other studies on perceived authenticity have so far focused on comparing formats such as SPs, video presentations, and text vignettes and different levels of authenticity within VPs [21]. Our study extends this literature by directly comparing SPs and VPs with respect to 3 frequently used perceived authenticity variables. This comparison seems particularly relevant, as both assessment formats are becoming increasingly popular. Our findings on the relationship between perceived authenticity and diagnostic accuracy are mixed. The equivalence test on correlations was not significant; therefore, we could not confirm the hypothesis that perceived authenticity is not meaningfully associated with diagnostic accuracy. However, a regular correlation between perceived authenticity and diagnostic accuracy that was calculated afterward was close to 0. Taken together, these findings of nonequivalence and nonsignificance indicate that we did not have sufficient power to draw a conclusion [45]. Nevertheless, we have found some indication that the correlation between perceived authenticity and diagnostic competences is rather small. This finding is in accordance with literature reviews [23,24], which reported small correlations between perceived authenticity and performance.

With regard to cognitive load, we found that the combined score is equivalent for SPs and VPs that use the same clinical cases. This finding substantiates the literature suggesting that cognitive load depends mainly on task complexity [29]. Moreover, the fact that the extraneous load was equivalent for SPs and VPs indicates that user interaction through a software menu does not substantially increase cognitive load. This finding is important because decreasing the cognitive load by allowing for user input using natural language processing [21] is still highly expensive. Our study also adds to the literature that the level of cognitive load is similar in SPs and VPs as assessment methods if the different types of cognitive load are systematically controlled for during the design process. In addition, we demonstrated that intrinsic and extraneous cognitive loads correlate negatively with diagnostic accuracy. The finding on intrinsic cognitive load corroborates that the interplay between materials and the assessed person’s expertise is associated with performance. The finding on extraneous cognitive load shows that unnecessary characteristics of the assessment environment can strain memory and attention and be detrimental to performance in assessment settings. Together, these findings fit well with the literature, which has repeatedly reported negative effects of intrinsic and extraneous cognitive loads on complex problem solving in medical education [27] and other domains [51]. Our study unveils that a negative relationship between intrinsic and extraneous cognitive loads and performance in a simulation-based measure of diagnostic competences already shows when overall cognitive load is medium on average.

Our study found no evidence that diagnostic accuracy was equivalent for SPs and VPs. In contrast, higher diagnostic accuracy was achieved for SPs than for VPs. The small number of studies comparing both assessment methods so far [1,31,32] have reported medium correlations, not taking into account different case content or testing time. Using the TOST procedure as a novel methodological approach, our study contributes to the literature by finding that grading was not equivalent, as participants received a better hypothetical grade when the simulation-based assessment was administered with SPs than with VPs. On the one hand, we cannot rule out that this finding may be explained by additional support from the actors in the SP assessment. To avoid and mitigate such an effect, actors were trained by an acting coach and a physician, memorized their symptoms and scripts, and did not know the diagnosis of their case. Moreover, student assistants screened all SP assessments, and no additional systematic support by actors was discovered. On the other hand, this finding can be explained by the lower appraisal of motivational value and the lower quantity of evidence generation reported for VPs. Participants solving VP cases may thus have been less engaged and may have collected a smaller number of important diagnostic cues that supported their diagnostic process.

Contrary to our expectations, the quality of evidence generation was not positively correlated with the *combined* diagnostic accuracy score. Closer inspection of the data revealed that the quality of evidence generation was positively correlated with diagnostic accuracy in *VPs*. This confirmed relationship is in line with the theoretical assumptions of Heitzmann et al [10]. In *SPs*, however, the quality of evidence was not correlated with diagnostic accuracy. This finding contradicts the theoretical assumptions of Heitzmann et al [10] and empirical results from studies using observational checklists with SPs [34] and real patients [36]. There are 2 explanations for these conflicting findings. First, the quality of evidence generation was, as an exploratory follow-up *t* test indicated, higher in VPs than in SPs. This higher quality of evidence generation could have been caused by a slightly different process of history taking in both assessment methods. Participants working with VPs selected questions from a menu. In contrast, participants working with SPs formulated questions during history taking freely. Second, SPs could have offered additional support to assessed persons who displayed a low quality of evidence generation, whereas VPs reacted in a completely standardized way to all assessed persons.

### Limitations

One methodological limitation of our study might be the low statistical power for the analysis of hypothesis H1.2 and related post hoc analyses that addressed the relationship between the perceived authenticity variables and diagnostic accuracy. This lack of statistical power can primarily be attributed to our investigation of whether a correlation of *r*=±0.20 or more extreme exists. As recommended by Lakens [46], the smallest effect size of interest was selected based on findings from the literature. Specifying the smallest effect size of interest to be larger would have increased power but not have contributed findings from a valuable equivalence test to the literature. This is the case because the literature already assumes a small effect size [23,24].

One theoretical limitation of the study is that the results on perceived authenticity may not generalize without restrictions to other related concepts of authenticity. Shaffer et al [15] argue that thick authenticity consists of four different aspects. An authentic task, situation, or material should (1) exist in real life, (2) be meaningful, (3) allow the learner to engage in professional activities of the discipline, and (4) be conducted rather similar in instruction and assessment. The authors assume that thick authenticity can only be achieved when all aspects of authenticity are adequate and that VPs could potentially achieve similar authenticity to SPs. Hamstra et al [16] proposed distinguishing fidelity using the terms physical resemblance and functional task alignment. The authors report weak evidence for the relationship between physical resemblance and performance, and strong evidence for the relationship between functional task alignment and performance. In our study, the concepts of thick authenticity and fidelity were not measured for two reasons. First, these concepts can, to some extent, only be judged externally by experts. Second, the repeated measures design of the study forced us to keep aspects such as thick authenticity, physical resemblance, and functional task alignment as similar as possible in SPs and VPs. Nevertheless, we believe that the relationship between different authenticity concepts and diagnostic competences still requires further research. Future studies should attempt to untangle the relationship between different authenticity concepts and diagnostic competences by measuring these systematically.

### Conclusions

Our findings on the relationship between perceived authenticity and diagnostic accuracy contribute to the debate on the costs and benefits of perceived authenticity in performance-based assessments. These results relativize the importance of perceived authenticity in assessment. Increasing the perceived authenticity of assessment methods above a certain necessary threshold and thus raising their costs [23] does not seem to be of much benefit. Such spending could potentially squander a large share of the medical education budget [52] that could be put to more valuable use. Our results on cognitive load highlight its importance as a process variable in assessment settings. Performance-based assessment should thus attempt to reduce extraneous load and control for intrinsic load to measure performance in a standardized way that is still close to clinical practice [53].

Finally, the findings on diagnostic competences have some practical implications if VPs are used as an alternative to SPs in assessment. In particular, we found that VPs could lead to lower diagnostic accuracy scores than SPs, which could, in turn, negatively affect students’ grades. There are 2 different mechanisms that could explain this finding: assessment with SPs could overestimate true performance or assessment with VPs could underestimate true performance. In accordance with SPs overestimating performance, we could not rule out additional support from the actors. In fact, the low, nonsignificant correlation between the quality of evidence generation and diagnostic accuracy in SPs, together with the higher diagnostic accuracy in SPs, could indicate that actors provided some additional support (eg, to participants who displayed low quality of evidence generation). Careful training [54] and screening thus seem to be of great importance to avoid additional support from actors during SP assessment to match the high level of standardization that VPs provide. The mechanism of possible underestimation of performance with VPs could be substantiated by the lower motivational value and quantity of evidence generation discovered for VPs. We suggest taking the following measures: students could be motivated additionally in VP assessment by more interactive environments (eg, using natural language processing) or providing automated elaborated feedback directly after the assessment. Moreover, the assessment time can be extended when menu-based VPs are used in practice. This way, the quantity of evidence generation could be raised to a level similar to that in the SP assessment.

## Appendix

Multimedia Appendix 1 Participant characteristics across all conditions and CONSORT (Consolidated Standards of Reporting Trials)–style diagram of participant flow.

Multimedia Appendix 2 Overview of the experimental procedure and simulation phases.

Multimedia Appendix 3 Table containing the questions provided with all virtual patients. These questions were allocated to the five history-taking categories of main symptoms, prior history, allergies and medication, social and family history, and system review.

Multimedia Appendix 4 Authenticity scales, cognitive load scales, coding scheme for diagnostic accuracy, coding scheme for the quality of evidence generation, motivation scales, and details of the diagnostic knowledge tests.

Multimedia Appendix 5 Boxplots and bee swarm plots for authenticity, cognitive load, and clinical reasoning variables for standardized patients and virtual patients.

## Abbreviations

CG: case group
NHST: null hypothesis significance testing
SP: standardized patient
TOST: 2 separate 1-sided test
VP: virtual patient
