# Supplementary material for: Assessment of Diagnostic Competences With Standardized Patients Versus Virtual Patients: Experimental Study in the Context of History Taking
Source: J Med Internet Res. 2021 Mar 4;23(3):e21196. doi: 10.2196/21196 (PMC7974754; doi:10.2196/21196)
Supplement: Multimedia Appendix 1 [file jmir_v23i3e21196_app1.docx]

**Multimedia Appendix 1.** Participant characteristics across all conditions and CONSORT (Consolidated Standards of Reporting Trials)–style diagram of participant flow.

Table 1

Participant characteristics across all conditions.

|  | Condition 1A | Condition 1B | Condition 2A | Condition 2B |
| --- | --- | --- | --- | --- |
|  |  |  |  |  |
| Age in years mean, (SD) | 25.42 (3.63) | 26.52 (4.45) | 26.71 (2.92) | 25.52 (6.63) |
| **Sex, n (%)** |  |  |  |  |
| Females | 11 (58) | 11 (52) | 14 (67) | 18 (72) |
| Males | 8 (42) | 10 (48) | 7 (33) | 7 (28) |
| **Expertise, n (%)** |  |  |  |  |
| Novices | 10 (53) | 12 (57) | 11 (52) | 14 (56) |
| Intermediates | 9 (47) | 9 (43) | 10 (48) | 11 (44) |

Assessed for eligibility (n=93)

Analysed (n=19)
♦ Excluded from analysis (insufficient language skills, technical difficulties) (n=4 )

## Analysis

## Allocation

## Enrollment

Excluded (n=0)

♦  Not meeting inclusion criteria (n=0)

♦  Declined to participate (n=0)

♦  Other reasons (n=0)

Randomized (n=93)

Allocated to intervention 2B (n= 25)

♦ Received allocated intervention (n= 25)

♦ Did not receive allocated intervention (give reasons) (n= 0)

Allocated to intervention 2A (n=22)

♦ Received allocated intervention (n=22)

♦ Did not receive allocated intervention (give reasons) (n= 0)

Allocated to intervention 1B (n=23)

♦ Received allocated intervention (n=23)

♦ Did not receive allocated intervention (give reasons) (n=0)

Allocated to intervention 1A (n=23)

♦ Received allocated intervention (n=23)

♦ Did not receive allocated intervention (give reasons) (n=0)

Analysed (n=24)
♦ Excluded from analysis (technical difficulties) (n=1)

Analysed (n=22)
♦ Excluded from analysis (n=0)

Analysed
 (n= 21)
♦ Excluded from analysis (technical difficulties) (n=2)
