# Supplementary material for: Assessment of Diagnostic Competences With Standardized Patients Versus Virtual Patients: Experimental Study in the Context of History Taking
Source: J Med Internet Res. 2021 Mar 4;23(3):e21196. doi: 10.2196/21196 (PMC7974754; doi:10.2196/21196)
Supplement: Multimedia Appendix 2 [file jmir_v23i3e21196_app2.docx]

**Multimedia Appendix 2.** Overview of the experimental procedure and simulation phases.

Table 1

Overview of the Experiment

| Part of the experiment | Activity / test | Duration in minutes |
| --- | --- | --- |
| Pretest |  |  |
|  | Briefing | 10 |
|  | Conceptual knowledge test | 40 |
|  | Strategic knowledge test | 40 |
| Break |  | 10 |
| Simulation phase I (Case 1- 3) |  | 70 |
| Break and change of modality |  | 5 |
| Simulation phase II (Case 4-6) |  | 70 |
| Debriefing/posttest |  |  |
|  | Working memory test | 20 |

Table 2

Overview of the Simulation Phases

| Part of the experiment | | Activity / test | | Duration in minutes |
| --- | --- | --- | --- | --- |
| Simulation briefing |  | | | 10 |
|  | | Fiction contract, | |  |
|  | | Familiarization with content | |  |
|  | | Familiarization with technical aspects | |  |
|  | | Interest scales | |  |
|  | | Motivation questionnaires | |  |
| Simulation phase  (similar for all three cases) | |  | | 60 |
|  | | Presentation of chief complaint | |  |
|  | | Independent history taking | |  |
|  | | Diagnostic accuracy measurement | |  |
|  | | Authenticity scales | |  |
|  | | Cognitive load scales | |  |
|  | | Epistemic emotions scales |  | |
