# Supplementary material for: Assessment of Diagnostic Competences With Standardized Patients Versus Virtual Patients: Experimental Study in the Context of History Taking
Source: J Med Internet Res. 2021 Mar 4;23(3):e21196. doi: 10.2196/21196 (PMC7974754; doi:10.2196/21196)
Supplement: Multimedia Appendix 3 [file jmir_v23i3e21196_app3.docx]

**Multimedia Appendix 3.** Table containing the questions provided with all virtual patients. These questions were allocated to the five history-taking categories of main symptoms, prior history, allergies and medication, social and family history, and system review.

| **NR** | **Main symptoms[HS]** | **Prior history [MV]** | **Allergies and medication [AM]** | **Social and family history [SF]** | **Systems review [SUE]** |
| --- | --- | --- | --- | --- | --- |
| 01 | Are you experiencing the complaints for the first time? | Do you know of any pre-existing conditions? | Do you have common or chronic infections for which you have to take antibiotics? | Did your parents or another one of your closer relatives die at a very young age? | Have you gained or lost any weight in recent weeks? |
| 02 | Do you suffer from pain? | Have you ever had surgery? | Do you take medication on a regular basis? Or do you maybe take special medication in specific situations? Something like medication for allergies or painkillers? | Has anyone in your family died a sudden cardiac death? | Do you have night sweats? |
| 03 | How are you doing right now? | Have you had surgery in recent weeks? | Have you noticed whether your eyes were twitching or your nose was running? | Do you smoke or did you use to smoke? | Have you eaten and drunk enough today? |
| 04 | Do you experience the complaints only during exertion or also at rest? | Has your mobility been limited, for instance by a plaster cast or through a disease that confined you to bed? | Do you have known allergies or asthma? | How much alcohol do you drink normally? | Have you noticed whether you were able to perform as well as usual? |
| 05 | Have you recently avoided leaving the house because you were afraid that something might happen? | Do you have a coagulation disorder? |  | Did you drink a lot of alcohol yesterday? | Are you sleeping well? |
| 06 | Did it occur suddenly or did you already experience dyspnea in recent days or weeks? | Have you had thrombosis before? |  | Have you completed occupational training? If so, for which occupation? | Do you have problems with your stool or with your urination? |
| 07 | Do you have a cough? | Do you suffer from high blood pressure? |  | Are you currently employed or do you own your own business? | Have you had bloody tarry stool or have you vomited blood recently? |
| 08 | Can you rest on a straight surface? | Have you had problems with your heart before? |  | Are you married? | Have you had fever or chills in the last few days? |
| 09 | Have you experienced occasional dizziness in the last few months? Or have you passed out? | Have you made use of psychotherapeutic treatment before? |  | Do you have children? | Do you have lip herpes at the moment? |
| 10 | Have you experienced fear of death? | Did you suffer from heart muscle inflammation as a child? |  | Do your parents or siblings have chronic diseases (e.g. high blood pressure, diabetes etc.)? | Do you have any pain in your arms or in the jaw area? |
| 11 | Have you experienced other symptoms? For instance, rapid heartbeat or dizziness? [Penultimate position within the category, excluded from analysis as it was only included in the “aimueller” case] | Do you suffer from a muscular disorder? |  | Have you taken a longer plane, bus, or car trip recently? | Do you feel any traction or tingling in your hands? |
| 12 | Were you chewing gum when it happened?  [Very last position within the category, excluded from analysis as it was only included in the “aimueller” case] | Have your thyroid glands been checked for overactivity or underactivity? |  | Do you exercise regularly? | Have your legs gotten bigger? |
| 13 |  | Have you had an acute infection in the last few weeks? Have you had a cough or cold or something similar? |  | Have you taken drugs or an energy booster or something similar recently? | Did you had the feeling of a racing or stumbling heart in the past few days? |
| 14 |  | Do you go for regular check-ups with your GP? |  | Can you tell me how much you usually drink per day? | What does your urine look like? Have you noticed any unusual colour? Extremely bright, dark or brown or anything like that? |
| 15 |  | Do you know how high your blood pressure is usually? |  | Do you have siblings? |  |
| 16 |  | Do you know whether any blood levels have been bad before? Cholesterol or similar values? |  | Are there any known hereditary diseases in your family? |  |
| 17 |  | Have you had a malignant illness before (e.g. cancer or a tumor or something similar)? |  | Have you been under a lot of stress recently? [last position within the category, excluded from analysis as it was only included in some cases] |  |
| 18 |  | Have you ever had a stroke? |  |  |  |
| 19 |  | Have you been treated by a neurologist before? |  |  |  |
| 20 |  | Have you ever had a pneumothorax or have you ever had lung surgery? |  |  |  |
| 21 |  | Are you pregnant or have you given birth recently?  [Last position within the category, excluded from analysis as it was only included in one case] |  |  |  |
