# Supplementary material for: Assessment of Diagnostic Competences With Standardized Patients Versus Virtual Patients: Experimental Study in the Context of History Taking
Source: J Med Internet Res. 2021 Mar 4;23(3):e21196. doi: 10.2196/21196 (PMC7974754; doi:10.2196/21196)
Supplement: Multimedia Appendix 4 [file jmir_v23i3e21196_app4.docx]

**Multimedia Appendix 4.** Authenticity scales, cognitive load scales, coding scheme for diagnostic accuracy, coding scheme for the quality of evidence generation, motivation scales, and details of the diagnostic knowledge tests.

Table 1

Authenticity scales

| Nr | Item |
| --- | --- |
| 1  2  3  4  5  6  7  8  9  10 | I consider the history-taking simulation as authentic.  The simulation of the medical interview seemed like a real professional demand.  The experience in the history-taking simulation resembled the experience of a real professional demand.  When I participated in history-taking, it seemed to me as if I was a real part of the simulated situation.  When I participated in history-taking, I felt like I was physically present in the clinical environment.  When I participated in history-taking, it seemed to me as if I could affect things, like in a real medical interview.  When I participated in history-taking, I focussed strongly on the situation.  When I participated in history-taking, I forgot intermittently that I take part in a study.  When I participated in history-taking, I was immersed in the situation.  When I participated in history-taking, I was fully engaged. |

Note. Items 1-3 measure the subscale realness, items 4-6 spatial presence and items 7-10 presence. This measure was used as a 5-point scale, ranging from 1 (“Strongly disagree”) to 5 (“Strongly agree”).

Table 2

Cognitive load scales

| Nr | Item |
| --- | --- |
| 1  2  3  4  5 | How easy or difficult do you consider „History taking for dyspnea" at this moment?  How easy or difficult is it for you to work with the simulation?  How easy or difficult is it for you to distinguish important and unimportant information in the simulation?  How easy or difficult is it for you to collect all the information that you need in the learning environment?  How easy or difficult was it to understand the last case? |

Note. Item 1 measures intrinsic load, items 2-4 extraneous load and item 5 germane load. This measure was used as a 5-point scale, ranging from 1 (“easy”), 2 ("rather easy"), 3 ("neutral"), 4 ("rather hard"), and 5 ("hard").

Table 3

*Coding Scheme for Diagnostic Accuracy for All Cases*

| Case | 1 Point (Fully correct) | 0.5 Points (Partially correct) | 0 Points (Incorrect) |
| --- | --- | --- | --- |
| CGA_1 | - Pulmonary embolism with lymphoma  - Pulmonary embolism with prostate cancer | - Pulmonary embolism  - Myelodysplastic syndrome | All other diagnoses from the long-menu |
| CGA_2 | - Congestive heart failure with atrial fibrillation  - Congestive heart failure with arrhythmia | - Left-sided heart failure  - Acute decompensated heart failure  - Right-sided heart failure  - Dyspnea caused by pleural effusion - [Acute coronary syndrome](https://www.linguee.co.uk/english-german/translation/acute+coronary+syndrome.html) | All other diagnoses from the long-menu |
| CGA_3 | - Hyperventilation tetany caused by panic attack  - Hyperventilation tetany caused by panic disorder | - Panic attack  - Panic disorder | All other diagnoses from the long-menu |
| CGB_1 | - Pulmonary embolism with coagulation disorder  - Pulmonary embolism with antiphospholipid syndrome  - Pulmonary embolism with  hereditary thrombophilia | - Pulmonary embolism | All other diagnoses from the long-menu |
| CGB_2 | - Community-acquired pneumonia (CAP)  -Bacterial pneumonia | - Acute bronchitis  - Pneumonia | All other diagnoses from the long-menu |
| CGB_3 | - Obstructive hypertrophic cardiomyopathy - Cardiac insufficiency with concentric left ventricular hypertrophy | - Cardiac insufficiency  - Right-sided heart failure  - Left-sided heart failure  - Valvular heart disease  - Dilated cardiomyopathy  - Arrhythmogenic right ventricular cardiomyopathy  - Myocarditis - Cardiomyopathy - Hypertrophic cardiomyopathy | All other diagnoses from the long-menu |

*Note.* Points allocated were defined as follows. 1 Point: A solution determined ex-ante by the author was discovered (i.e., one type of the disease for which the case was created was listed). 0.5 Points: The listed diagnosis can be explained by the symptoms and clinical findings (e.g., from prior information and replies of the patient). 0 Points: An incorrect diagnosis is listed, a diagnosis with incorrect additional information is listed or no diagnosis is listed.

Table 4

*Coding Scheme for the Quality of Evidence Generation*

| CGA_1 | CGA_2 | CGA_3 | CGB_1 | CGB_2 | CGB_3 |
| --- | --- | --- | --- | --- | --- |
| HS02 | HS02 | HS03 | MV01 | HS02 | HS02 |
| SUE01 | HS03 | HS05 | MV05 | HS03 | HS09 |
| SUE02 | SUE06 | HS09 | SF16 | MV13 | MV07 |
| SUE04 | SUE13 | HS10 | HS02 | SUE02 | AM02 |
| SUE05 | SUE14 | SUE01 | HS03 | SUE04 | SF01 |
| SUE06 | SF14 | SUE05 |  | SUE08 | SF02 |
| SUE14 |  | SUE11 |  |  | SF12 |
|  |  | SUE13 |  |  | SUE01 |
|  |  |  |  |  | SUE04 |
|  |  |  |  |  | SUE05 |
|  |  |  |  |  | SUE12 |
|  |  |  |  |  | SUE13 |

Note. Only items highly relevant for the case are marked with the corresponding code.

The abbreviation CG corresponds to the case-group, the number to the case number.

Table 5

*Motivation scales*

| Nr | Item |
| --- | --- |
| 1  2  3  4  5  6  7  8 | I believe I am up to the difficulty of this task.  I will probably not solve this task successfully (Reverse coded)  I believe everyone can solve this task.  Probably, I will not solve this task successfully (Reverse coded).  I believe it is important to be able to solve this task.  Even if this task is not part of examinations, it is important to be able to solve this task.  It would be useful to engage oneself with this task.  It would be useful to occupy oneself with this task, as it is generally useful to master this type of task. |

Note. Items 1-4 measure the expectancy aspect of motivation, items 5-8 the value aspect of motivation. The scale for the expectancy aspect ranged from (1) strongly disagree to (7) strongly agree. The scale for the value aspect ranged from (1) strongly disagree to 5 strongly agree. The instruction used was: "We would like to know more about your current attitude towards the presented task. Please indicate the attitude that suits best to you."

Table 6

Details of the diagnostic knowledge tests

|  | Conceptual knowledge | Strategic knowledge |
| --- | --- | --- |
| Content | 40 multiple-choice questions on dyspnea with the answer formats single-choice and Pick-N | 10 case vignettes on dyspnea with four single-choice questions each |
| Reliability | Cronbach α=.76. | Cronbach α=.81 |
| Scoring | In single-choice questions, participants received 1.0 points for selecting the correct answer and 0 points for an incorrect answer. In Pick-N questions, participants were allocated 1.0 points for each entirely correct answer pattern and partial credit (0.50 points) if they provided at least 50 percent of the correct answers to a question. | |
| Total score | We divided the number of correct answers by the number of provided questions to calculate scores for the knowledge tests. Thus, both knowledge test scores ranged from 0 to 1. | |
