# Supplementary material for: Assessment of Diagnostic Competences With Standardized Patients Versus Virtual Patients: Experimental Study in the Context of History Taking
Source: J Med Internet Res. 2021 Mar 4;23(3):e21196. doi: 10.2196/21196 (PMC7974754; doi:10.2196/21196)
Supplement: Multimedia Appendix 5 [file jmir_v23i3e21196_app5.docx]

**Multimedia Appendix 5.** Boxplots and bee swarm plots for authenticity, cognitive load, and clinical reasoning variables for standardized patients and virtual patients.

Figure 1. Boxplots and beeswarmplots of authenticity variables in standardized patients and virtual patients. Means are highlighted with a dot in the box. Within beeswarmplots points visualize the distribution of the variable similar to a scatterplot.


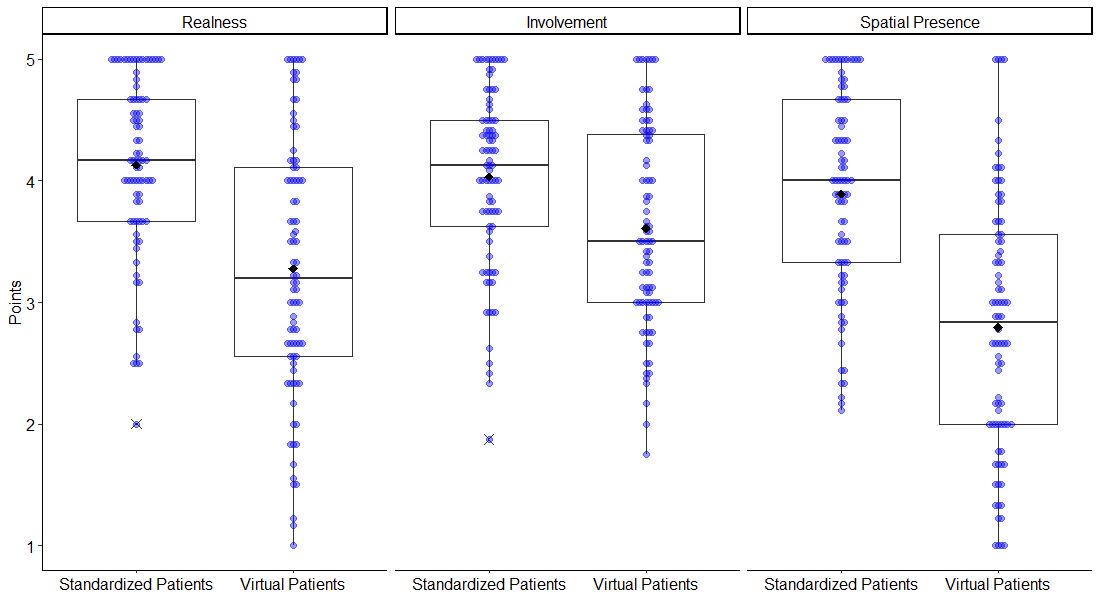


Figure 2. Boxplots and beeswarmplots of cognitive load variables in standardized patients and virtual patients. Means are highlighted with a dot in the box. Within beeswarmplots points visualize the distribution of the variable similar to a scatterplot.
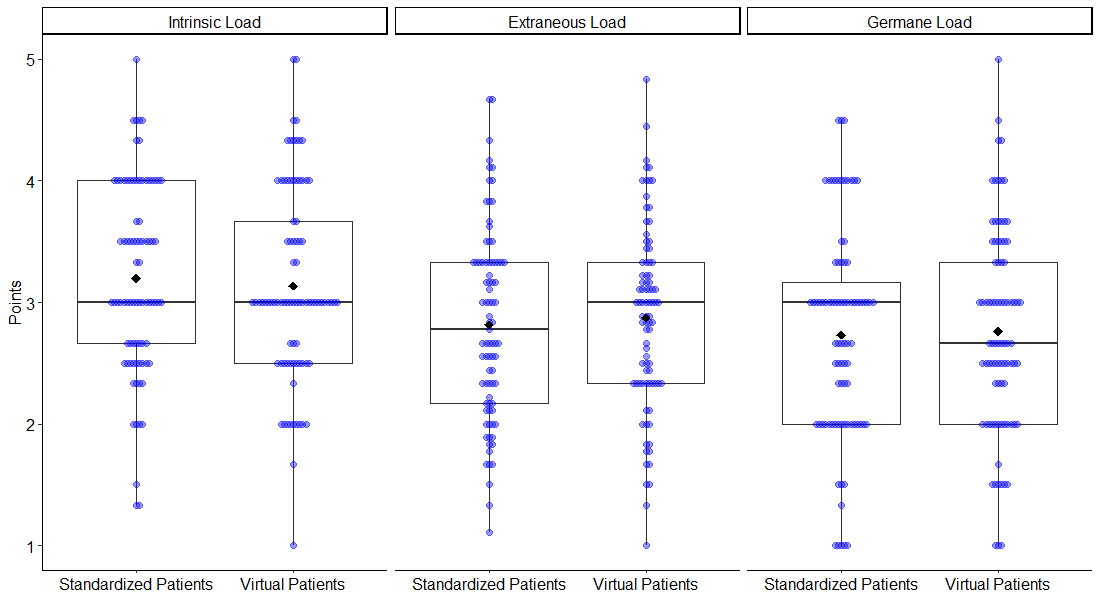


Figure 3. Boxplots and beeswarmplots of clinical reasoning variables in standardized patients and virtual patients. Means are highlighted with a dot in the box. Within beeswarmplots points visualize the distribution of the variable similar to a scatterplot.


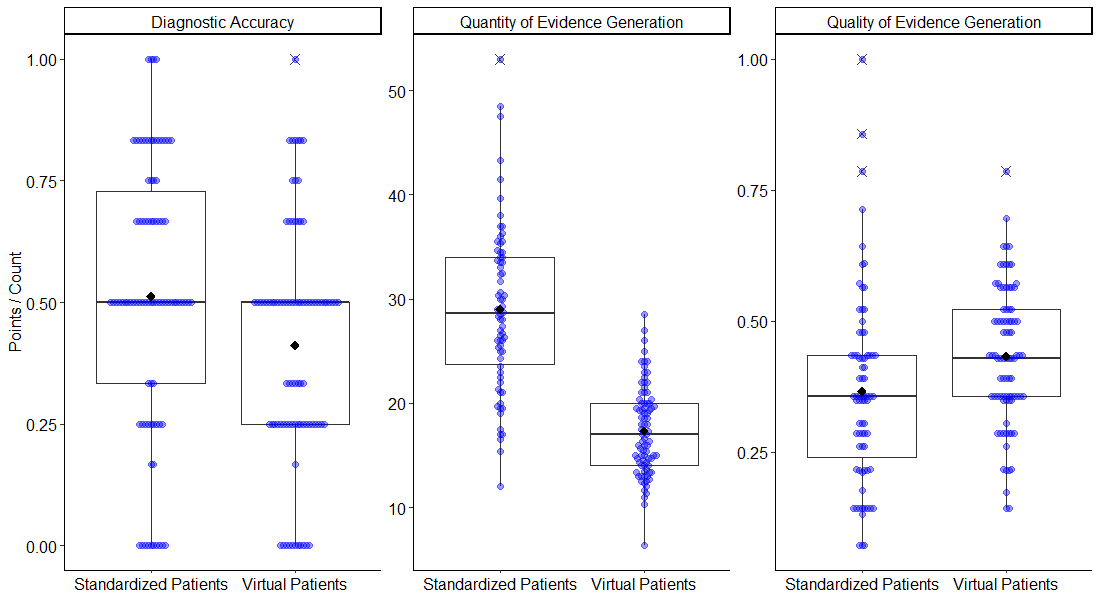


Note

The R-packages ggplot and ggebeeswarm were utilized for data visualization.
